# Supplementary material for: Top-DTI: integrating topological deep learning and large language models for drug–target interaction prediction
Source: Bioinformatics. 2025 Jul 15;41(Suppl 1):i133–41. doi: 10.1093/bioinformatics/btaf183 (PMC12261471; doi:10.1093/bioinformatics/btaf183)
Supplement: btaf183_Supplementary_Data [file btaf183_supplementary_data.pdf]

**Top-DTI: Integrating Topological Deep Learning and Large Language Models for Drug Target Interaction Prediction**

Muhammed Talo and Serdar Bozdag

# Supplementary Document

## Baseline and SOTA Methods

We evaluated the performance of the Top-DTI model by comparing it with the following baseline and SOTA methods:

- DeepConv-DTI employs a CNN to extract local residue patterns from raw protein sequences and combines these features with drug fingerprints.
- GraphDTA model represents drugs as molecular graphs and utilizes a GNN to learn chemical features and interactions directly from the graph architecture.
- MolTrans uses a frequent consecutive subsequence (FCS) mining algorithm to extract substructures from proteins and drugs and then encodes them using a transformer-based architecture to predict DTI.
- DrugBAN integrated a GCN to encode local structures from drug molecular graphs with a CNN to encode protein sequences. Then, a bilinear attention network processes these encoded features for DTI prediction.
- DLM-DTI adopts the ChemBERTa model for drug feature extraction and the ProtBERT transformer for protein representations. These features are concatenated and used to train an MLP architecture to predict DTI.
- Kang et al. employ ChemBERTa and ProtBERT transformer-based models to encode drug molecules and protein sequences and implement prediction using a classifier.
- DrugLAMP uses a multimodal framework that combines molecular graph and protein sequence features extracted from the ESM-2 and ChemBERTa-2 LLMs. Pocket-guided co-attention and paired multimodal attention are applied to encode these features to predict DTI.

Supplementary Table S1: AUROC-Based evaluation of DTI models in low-resource training scenarios (AUROC)

| Training Data (%) | DeepDTA | DeepConv-DTI | MolTrans | **Top-DTI** |
|---|---|---|---|---|
| 30 | 0.838 ± 0.004 | 0.845 ± 0.003 | 0.853 ± 0.004 | **0.901 ± 0.003** |
| 20 | 0.821 ± 0.008 | 0.825 ± 0.003 | 0.832 ± 0.003 | **0.883 ± 0.002** |
| 10 | 0.787 ± 0.011 | 0.792 ± 0.004 | 0.802 ± 0.004 | **0.827 ± 0.004** |
| 5 | 0.762 ± 0.004 | 0.726 ± 0.008 | 0.768 ± 0.005 | **0.774 ± 0.006** |

As shown in Supplementary Table S1, Top-DTI consistently outperforms baseline methods, demonstrating greater robustness in data-scarce environments. At 5% training data setting, Top-DTI achieves an AUROC of 0.774. These findings underscore Top-DTI's capacity to generalize from limited labeled data, making it especially advantageous for practical drug development applications, where experimental data is usually limited.

Supplementary Table S2. The validation of top predicted drug–target interactions identified by Top-DTI

| DrugBank ID | Drug Name | Protein (Uniprot ID) | Gene | Evidence |
|---|---|---|---|---|
| DB04903 | Pagoclone | Q13936 | CACNA1C | CACNA1C is associated with anxiety and mood disorders; pagoclone modulates related pathways [1]. |
| DB00402 | Eszopiclone | P50406 | HTR6 | HTR6 was predicted as a common target of tetradecanal, a JTW compound related to sleep regulation [2]. |
| DB01019 | Bethanechol | P04798 | CYP1A1 | CYP1A1 is involved in clonidine metabolism, and bethanechol and clonidine are listed among active pharmaceutical ingredients in the same regulatory context [3]. |
| DB03044 | Doramapimod | Q02750 | MAP2K1 | Doramapimod targets JNK and p38 signaling; MAP2K1 was significantly upregulated in fulvestrant-resistant cells [4]. |
| DB01620 | Pheniramine | P14867 | GABRA1 | GABRA1, encoding a GABA-A receptor subunit, is highlighted as a candidate gene in GABAergic signaling, a pathway implicated in depression [5]. |

Supplementary Table S3: Ablation Results for GNN and Static Fusion Variants on Unseen Drug and Unseen Target Datasets.

| | Unseen Drug | | Unseen Target | |
|---|---|---|---|---|
| | **AUROC** | **AUPRC** | **AUROC** | **AUPRC** |
| Betti Fusion | 0.907 ± 0.002 | 0.922 ± 0.002 | 0.902 ± 0.002 | 0.898 ± 0.002 |
| Betti Static Fusion | 0.905 ± 0.002 | 0.919 ± 0.002 | 0.899 ± 0.005 | 0.895 ± 0.006 |
| PL Fusion | 0.908 ± 0.004 | 0.921 ± 0.004 | 0.903 ± 0.004 | 0.901 ± 0.003 |
| PL Static Fusion | 0.904 ± 0.004 | 0.918 ± 0.002 | 0.903 ± 0.002 | 0.900 ± 0.002 |
| Static Fusion | 0.907 ± 0.005 | 0.921 ± 0.004 | 0.906 ± 0.004 | 0.904 ± 0.006 |
| Without GNN | 0.887 ± 0.006 | 0.903 ± 0.004 | 0.853± 0.009 | 0.857± 0.007 |
| Top-DTI | 0.911 ± 0.003 | 0.924 ± 0.002 | 0.907 ± 0.003 | 0.904 ± 0.003 |

As reported in Supplementary Table S3, the dynamic fusion mechanism demonstrates marginally superior performance relative to the static fusion in both the Betti and PL feature configurations, underscoring the advantages of dynamic weighting in effectively integrating topological and LLM features. Additionally, to investigate the impact of GNN in Top-DTI, we performed an ablation study in which the feature fusion output was directly fed into an MLP without employing the GNN. Top-DTI yields better performance with GNN for both unseen drug and unseen target datasets.

This shows that using relational information from the DTI graph structure enhances prediction accuracy.

**Dynamic weighting of the feature fusion module:**

The TOP-DTI model assigns greater weights to LLM-based embeddings, 72% for drugs, and 64% for targets, as shown in Supplementary Figure S1. An increased alpha value signifies the model's dependence on LLM embeddings. Initially, alpha values were approximately 0.50, giving equal weight to LLM and topological features, similar to Static Fusion. As training progresses, alpha values were updated dynamically, rising to approximately 0.72 for drugs and 0.64 for targets. This indicates that the model effectively prioritized LLM embeddings over topological features by giving a weight of only 0.28 and 0.36 for topological features of drugs and targets, respectively. In situations where a modality provides limited information, such as ambiguous contact maps, the dynamic mechanism facilitates flexible reweighting, in contrast to Static Fusion, which lacks adaptability. This adaptive behavior results in consistent performance improvements in dynamic fusion models.

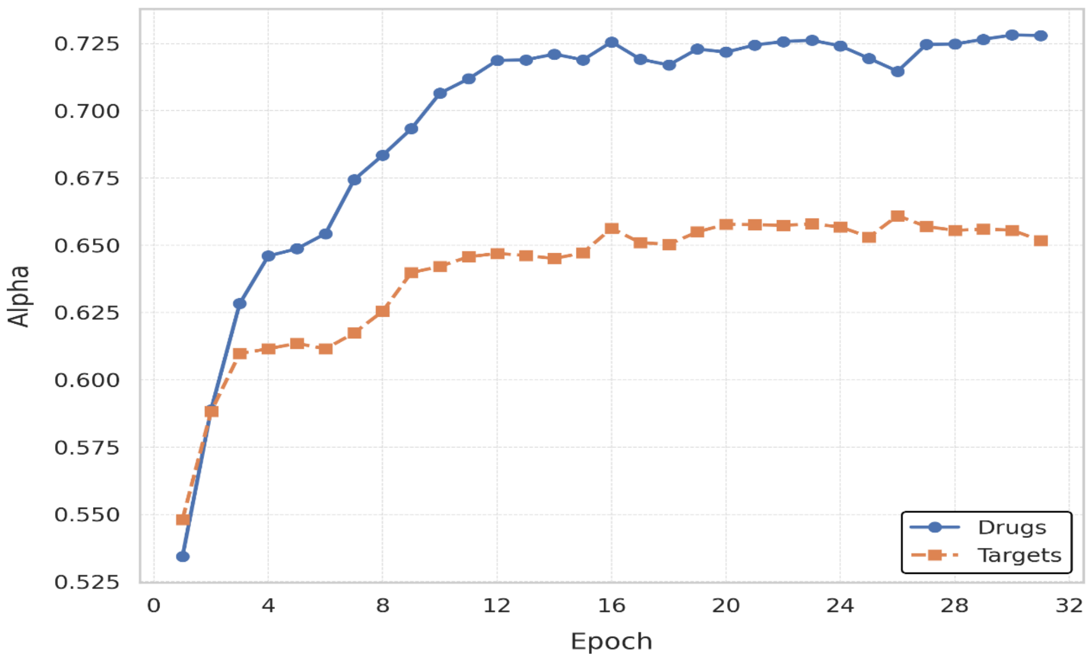


**Supplementary Figure S1.** Mean alpha values for drugs and targets during training.

## Statement on LLM Usage

We used LLMs only for grammar correction, spell-checking, and code debugging. All text in our study was written by us, with no content generated by LLMs included.

## References

1. Dao, D. T., Mahon, P. B., Cai, X., Kovacsics, C. E., Blackwell, R. A., Arad, M., ... & Bipolar Genome Study (BiGS) Consortium. (2010). Mood disorder susceptibility gene CACNA1C modifies mood-related behaviors in mice and interacts with sex to influence behavior in mice and diagnosis in humans. *Biological psychiatry*, *68*(9), 801-810.
2. Liu, X., Yuan, Z., Zeng, C., Huang, Y., Xu, X., Guo, W., ... & Zhan, R. (2022). Role of the volatile components in the anti-insomnia effect of Jiao-Tai-Wan in PCPA-induced insomnia rats. *Clinical Complementary Medicine and Pharmacology*, *2*(1), 100023.
3. Parrish, R. H., Ashworth, L. D., Löbenberg, R., Benavides, S., Cies, J. J., & MacArthur, R. B. (2022). Compounded nonsterile preparations and FDA-approved commercially available liquid products for children: a North American update. *Pharmaceutics*, *14*(5), 1032.
4. Tian, P., Zheng, J., Qiao, K., Fan, Y., Xu, Y., Wu, T., ... & Wang, H. (2025). scPharm: identifying pharmacological subpopulations of single cells for precision medicine in cancers. *Advanced Science*, *12*(2), 2412419.
5. Bao, K. (2021). Identifying Potential Novel Pathways and Therapeutic Targets of Major Depressive Disorder with Functional Genomics.
